# Study on the Damage Characteristics of Wheat Kernels under Continuous Compression Conditions

**DOI:** 10.3390/foods13182981

**Published:** 2024-09-20

**Authors:** Xiaopeng Liu, Ziang Shi, Yonglin Zhang, Hui Li, Jin Zhou, Hongjun Yang

**Affiliations:** 1 School of Mechanical Engineering, Wuhan Polytechnic University, Wuhan 430023, China; 18772461221@163.com (Z.S.); yin248247640@163.com (Y.Z.); lihui09@whpu.edu.cn (H.L.); hongjun_yang@163.com (H.Y.); 2 School of Electrical and Electronic Engineering, Wuhan Polytechnic University, Wuhan 430023, China; zhoujin@whpu.edu.cn

**Keywords:** peeling process, wheat kernels, Hertz theory, elastic–plastic compression, continuous damage model

## Abstract

Peeling wheat yields higher-quality flour. During processing in a flaking machine, wheat kernels undergo continuous compression within the machine’s chamber. As this compression persists, damage to the kernels intensifies and accumulates, eventually leading to kernel breakage. To study the damage characteristics of wheat kernels during peeling, this study established a continuous damage model based on Hertzian contact theory and continuous damage theory. The model’s accuracy was validated through experiments, culminating in the calculation of critical parameters for wheat peeling. This study focused on different wheat varieties (Ningmai 22 and Jichun 1) and kernel sizes (the thicknesses of the small, medium, and large kernels were standardized as follows: Ningmai 22—2.67 ± 0.07 mm, 2.81 ± 0.07 mm, and 2.95 ± 0.07 mm; Jichun 1—2.98 ± 0.11 mm, 3.20 ± 0.11 mm, and 3.42 ± 0.11 mm). Continuous compression tests were conducted using a mass spectrometer, and critical damage parameters were analyzed and calculated by integrating the theoretical model with experimental data. The test results showed that the average maximum crushing force (*F_c_*) for small, medium, and large-sized kernels of Ningmai 22 was 96.71 ± 2.27 N, 110.17 ± 2.68 N, and 128.41 ± 2.85 N, respectively. The average maximum crushing deformation (α*_c_*) was 0.65 ± 0.08 mm, 0.68 ± 0.13 mm, and 0.77 ± 0.17 mm, respectively. The average elastic–plastic critical pressure (*F_s_*) was 50.21 N, 60.13 N, and 59.08 N, respectively, and the average critical values of elastic–plastic deformation (*α_s_*) were 0.37 mm, 0.38 mm, and 0.39 mm, respectively. For Jichun 1, the average maximum crushing force (*F_c_*) for small-, medium-, and large-sized kernels was 113.34 ± 3.15 N, 125.28 ± 3.64 N, and 136.15 ± 3.29 N, respectively. The average maximum crushing deformation (*α_c_*) was 0.75 ± 0.11 mm, 0.83 ± 0.15 mm, and 0.88 ± 0.18 mm, respectively. The average elastic–plastic critical pressure (*F_s_*) was 58.11 N, 64.17 N, and 85.05 N, respectively, and the average critical values of elastic–plastic deformation (*α_s_*) were 0.45 mm, 0.47 mm, and 0.52 mm, respectively. The test results indicated that during mechanical compression, if the deformation is less than *α*_s_, the continued application of the compression load will not result in kernel crushing. However, if the deformation exceeds *α_s_*, continued compression will lead to kernel crushing, with the required number of compressions decreasing as the deformation increases. If the deformation surpasses *α_c_*, a single compression load is sufficient to cause kernel crushing. Since smaller wheat kernels are more susceptible to breakage during processing, the peeling pressure (*F*) within the chamber should be controlled to remain below the average elastic–plastic critical pressure (*F_s_*) of small-sized wheat kernels. Additionally, the kernel deformation (*α*) induced by the flow rate and loading in the chamber should be kept below the average elastic–plastic critical deformation (*α_s_*) of small-sized wheat kernels. This paper provides a theoretical foundation for the structural design and optimization of processing parameters for wheat peeling machines.

## 1. Introduction

Wheat is one of the earliest crops cultivated by humans and can provide over 20% of the protein required by the human body, equivalent to the combined protein content of meat, eggs, and dairy products, highlighting its high nutritional value [1]. China is the world’s largest producer and consumer of wheat. In 2023, the wheat cultivation area was 23,627 thousand hectares, with a total output of 136.59 million tons and a total trade value exceeding CNY 319.6 billion. The production and processing of wheat play a significant role in the national economy [2]. Wheat must be processed into flour before it can be consumed, with the processing primarily divided into two main stages: cleaning and milling. The efficiency of each step in the milling process is crucial to the quality of the flour. Traditionally, milling involves grinding and breaking the cleaned wheat kernels directly, followed by separating the wheat bran from the flour through sieving, brushing, and clearing, which often results in flour with a relatively high bran content and lower quality [3,4,5]. To address these issues, the peeling and milling process has been increasingly adopted in high-quality flour production. This process involves removing some of the outer layers from cleaned wheat kernels before sieving, brushing, and clearing to separate any remaining bran from the flour, resulting in a higher quality product [6,7,8,9]. This method not only enhances flour quality but also reduces production costs and improves efficiency [10].

**Figure 1:**
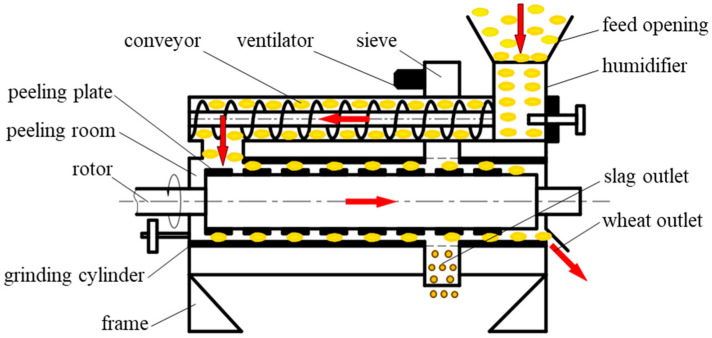
Wheat milling and peeling principle.

In the peeling powder-making process, a peeling machine is used to remove the surface skin from wheat kernels. The structure and working principle of the peeling machine are illustrated in Figure 1. During operation, wheat kernels undergo continuous extrusion inside the peeling chamber. This continuous pressure causes gentle grinding between the wheat and the peeling plate, as well as between individual kernels, allowing for the separation of the skin layer (bran) without damaging the kernels. The bran is expelled through a screen, while the dehulled wheat kernels are discharged from the machine for subsequent processing [11,12]. However, if the continuous extrusion pressure is too high, prolonged exposure can cause excessive damage to the wheat kernels, leading to breakage when the accumulated damage surpasses the plastic limit. After crushing, large-sized wheat kernels may have difficulty shedding the skin layer, resulting in increased bran content in the final product and failing to achieve the desired processing outcome. Additionally, small-sized kernels, which are crushed into powder, are discharged along with the bran, causing significant material loss. Therefore, the damage to and crushing of wheat kernels under continuous extrusion are crucial factors affecting the efficacy of the peeling and milling process. The structural and operational parameters of the peeling machine are key factors influencing the extrusion pressure inside the chamber. Due to the unclear damage mechanisms of wheat kernels under continuous extrusion conditions, the design and parameter optimization of peeling machines lack a reliable basis. This leads to issues such as incomplete peeling or excessive crushing and material loss during processing [13,14]. Thus, understanding the damage characteristics of wheat under continuous extrusion conditions is essential for the rational design of peeling machine structures and operational parameters. This knowledge will contribute to improving processing quality, reducing costs, and enhancing efficiency.

Research on the damage characteristics of wheat kernels provides valuable theoretical insights for improving wheat processing technologies, optimizing equipment parameters, reducing processing losses, and enhancing the quality of wheat products. Early studies in this area primarily focused on identifying the types of damage wheat kernels sustain. For instance, Chen ZP et al. investigated the resistance of wheat kernels to external forces through compression and friction tests. Their findings indicated that wheat kernels exhibit better resistance to abrasion than to extrusion [15]. Since wheat kernels are more prone to compression damage, subsequent research concentrated on factors affecting their compressive damage characteristics. Cheng X et al., for example, examined the influence of moisture content on the compression and crushing behavior of wheat kernels and proposed a model relating the compressive force to moisture content [16]. Zhang K et al. analyzed the compressive damage characteristics across different wheat varieties and concluded that the internal structure, shape, and size of the kernels play significant roles in determining their crushing load [17]. Similarly, Barrer GN et al. demonstrated, through uniaxial compression tests, that kernel stiffness, fracture force, and deformation are negatively correlated with moisture content [18]. These studies underscore the significant impact of factors such as moisture content and variety on the damage characteristics of wheat kernels. However, they do not provide an in-depth analysis of the entire damage process. To bridge this gap, later research shifted to investigating the damage evolution of wheat kernels and the changes in kernel characteristics throughout each damage stage. For example, Omarov A et al. studied the deformation and crushing behavior of wheat kernels using compression tests, and by analyzing force-deformation curves, they found that elliptical wheat kernels exhibit quasi-elastic and elastic–plastic deformation behaviors under applied force [19]. Ponce-García N et al. further identified that the damage process of wheat kernels includes a distinct elastic–plastic deformation phase, which is influenced by the kernel variety and moisture content. Kernels with a higher moisture content and softer texture displayed greater plasticity, while those with lower moisture content were more brittle [20,21]. Fernandes LS et al. also confirmed through mechanical testing that the compressive force decreases as the moisture content increases, while the proportional deformation modulus rises as the moisture content decreases, highlighting the relationship between water content and kernel deformation [22]. Although these studies have advanced our understanding of wheat kernel damage characteristics, their practical relevance to optimizing dehulling parameters remains limited. This is primarily because the experiments involved single compression tests, which do not accurately reflect the continuous extrusion and grinding that wheat kernels undergo during the dehulling process. Additionally, there has been no theoretical modeling of the elastic–plastic phase in wheat kernel compression.

In the actual wheat peeling and milling process, wheat kernel damage occurs under continuous loading, manifesting as progressive damage. Therefore, studying the continuous damage characteristics of wheat kernels is of great importance for optimizing the parameter regulation in the actual wheat peeling process. To date, no research has been conducted specifically on the continuous damage characteristics of wheat kernels. However, several scholars have studied the continuous damage processes and modeled them for other materials, such as rapeseed, brown rice, and walnuts. For instance, Yuan Jiacheng et al. applied continuous damage theory to establish a compression damage evolution equation for rapeseed kernels, analyzing the cumulative compression damage process through experimental validation [23]. Similarly, Liu X et al. used continuous damage theory to develop a continuous damage model for brown rice kernels, verifying the model through experiments and identifying the critical elastic–plastic deformation during compression as the initial state when the damage occurs [24]. Man XL et al. established and validated a damage model for walnut kernels, analyzing the effects of particle size and impact energy on the continuous damage process, and determining the relationship between the damage accumulation coefficient and material size [25]. These studies have successfully constructed continuous damage models for various kernels using continuous damage theory, which have been further verified and analyzed by scholars. For example, Tavares LM et al. investigated the influence of particle size and shape on continuous damage parameters and verified the accuracy of a continuum damage model for particle rupture under continuous loading conditions [26]. Han T et al. analyzed the fatigue strength of individual particles under continuous compression by establishing an ideal fatigue model, showing that particle fatigue strength decreases with increasing compression cycles before rupture occurs [27]. In summary, the existing research demonstrated that the continuous damage process of material particles results from the accumulation of single compression damages, and damage parameters change significantly as the load accumulates. While continuous damage theory has been widely applied to materials such as rapeseed, brown rice, and walnuts, no study has yet focused on establishing a continuous damage model for wheat kernels. Due to differences in species, shape, size, and other factors, the continuous damage processes and models for different materials vary, making models developed for other materials not entirely applicable to wheat kernels. Therefore, it is necessary to develop a specific continuous damage model tailored to wheat kernels.

This study addressed the aforementioned practical issues by investigating the continuous extrusion damage characteristics of wheat kernels. Through the analysis of damage accumulation and parameter changes during the continuous extrusion process, we developed a continuous damage model for wheat kernels. This model provides critical damage parameters, offering theoretical insights for optimizing the structure and operational parameters of wheat peeling machines and improving the effectiveness of peeling and processing technology.

## 2. Construction of a Continuous Damage Model for Wheat Kernels

There has been substantial research and analysis on the continuous damage characteristics of granular materials. For instance, Tavares used continuous damage theory and Hertzian contact theory to develop a theoretical model for continuous compression damage to spherical particles [26]. This model has been widely applied in studies of the continuous damage characteristics of conventional spherical granular materials, as shown in Equation (1):

(1) $$P=\frac{d^{0.5}}{3}\bar{k}\delta^{\frac{3}{2}}$$

where *P* is the compression load; *d* is the particle diameter; $\bar{k}$ is the effective stiffness; and *δ* is the compression deformation.

The effective stiffness $\bar{k}$ of the material properties of the particles is

(2) $$\bar{k}=\left(1-D\right)k$$

where *D* is the amount of compression damage of the particles and *k* is the initial stiffness.

The damage evolution equation for compression is defined as

(3) $$D={\left(\frac{\delta }{\delta_{c}}\right)}^{r}$$

where *δ_c_* is the maximum deformation of particles during compression crushing and *γ* is the damage index.

The Tavares continuous damage model indicates that external compression loads cause deformation in particles, leading to damage. However, according to Hertzian contact theory [28,29], if the particle contact deformation remains within the elastic deformation limit, the particles are in a state of complete elastic contact and will revert to their original form upon unloading, thus avoiding damage. This limitation suggests that the Tavares model has constraints in certain scenarios. Furthermore, the research by Omarov, A et al. revealed that ellipsoidal wheat kernels exhibit quasi-elastic and elastic–plastic deformation behaviors under external loads [19]. Therefore, the Tavares continuous damage model may not be fully applicable to wheat kernels due to their ellipsoidal shape and elastic–plastic properties. In this study, we developed a continuous damage model for wheat kernels, building on continuous damage theory and the Tavares model, and making necessary adjustments to account for the specific material properties and compression damage characteristics of wheat kernels.

### 2.1. Correction of Material Properties of Wheat Kernels

**Figure 2:**
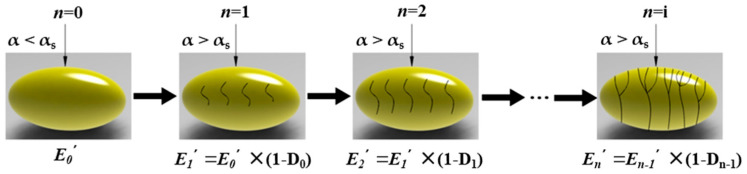
Continuous compression damage accumulation of a wheat kernel.

Ellipsoidal wheat kernels undergo both elastic and plastic deformation when subjected to successive external loads. During n successive compressions, the wheat kernels accumulate damage with each compression, resulting in changes to the elastic modulus after each compression, as illustrated in Figure 2.

Based on the actual performance behavior of these wheat kernels, the modulus of elasticity E_n_* after the nth compression was used in this study to replace the initial stiffness *k* in Equation (2), thereby correcting the initial material property parameters.

The maximum radius of curvature *R_n_*, the minimum radius of curvature $R_{n}^{\prime }$, and the directional angle *θ_n_* of the contact area when the wheat kernels are subjected to the nth compressive load were calculated as follows:

(4) $$R_{n}=\frac{L_{n}^{2}}{2H_{n}}$$

(5) $$R_{n}^{\prime }=\frac{B_{n}^{2}}{2H_{n}}$$

(6) $$cos\theta_{n}=\frac{\left(\frac{1}{R_{n}^{\prime }}-\frac{1}{R_{n}}\right)}{\left(\frac{1}{R_{n}^{\prime }}+\frac{1}{R_{n}}\right)}$$

where *L_n_*, *B_n_*, and *H_n_* are the length, width, and thickness of the wheat kernels after the nth compression, respectively.

The value of *cosθ_n_* can be obtained from the contact relationship tables in the ASAE S368.4 DEC2000 (R2017) standard [30] to calculate the dimensionless coefficient *K_n_*. Using the Hertz contact theory formula for ellipsoids, the apparent modulus of elasticity *E_n_* of the wheat kernels after the nth compression can be calculated as follows:

(7) $$E_{n}=\frac{3F{\left(1-\mu \right)}^{2}}{\pi \alpha^{\frac{3}{2}}}{\left[K_{n}{\left(\frac{1}{R_{n}^{\prime }}+\frac{1}{R_{n}}\right)}^{\frac{1}{3}}\right]}^{\frac{3}{2}}$$

where *F* is the normal load; *μ* is the Poisson’s ratio, and the wheat Poisson’s ratio takes a value of 0.42 [16]; and α is the compression deformation.

The modulus of elasticity E_n_* of wheat kernels after the nth compression is

(8) $$E_{n}^{*}=\frac{E_{n}}{1-\mu^{2}}$$

### 2.2. Correction for the Amount of Compression Damage in Wheat Kernels

When wheat kernels are in the elastic compression stage, the deformation is reversible and no kernel damage occurs. Once the compression deformation exceeds the elastic limit, the wheat undergoes plastic deformation and begins to exhibit damage. When the compression deformation reaches the maximum crushing deformation, the wheat is fully damaged and crushed. Assuming the damage of wheat kernels in the nth compression is D_n_*, and the elastic moduli of the wheat kernels in the (n − 1)th and nth compressions are *E_n_*_−1_* and E_n_*, respectively, these can be corrected using Formula (2):

(9) $$E_{n}^{*}=E_{n-1}^{*}\left(1-D_{n}^{*}\right)=E_{0}^{*}\overset{n}{\underset{i=1}{\prod }}\left(1-D_{i}^{*}\right)$$

The relationship between the modulus of elasticity E_n_* of the wheat kernels after the nth compression and the initial modulus of elasticity *E*_0_* of the wheat kernels is

(10) $$\{\begin{array}{c} E_{n}^{*}=E_{0}^{*}\;0<\alpha \leq \alpha_{s}\\ 0<E_{n}^{*}<E_{0}^{*}\;\alpha_{s}<\alpha <\alpha_{c}\\ E_{n}^{*}=0\;\alpha =\alpha_{c} \end{array}$$

where *α* is the compression deformation of the wheat kernels; *α*_s_ is the yield limit deformation; and *α_c_* is the maximum crushing deformation of the wheat kernels.

From Equations (9) and (10), it can be observed that after n consecutive compressions:

- If the compression deformation *α* is less than the yield limit deformation *α_s_*, then the damage variable D_n_* = 0, indicating no damage to the kernels.
- If the compression deformation *α* exceeds the yield limit deformation *α_s_*, then D_n_* > 0, and the kernels sustain damage.
- If the compression deformation *α* reaches the maximum crushing deformation *α*_c_, then D_n_* = 1, indicating that the kernels are completely damaged and crushed.

Based on the Tavares continuous damage model, Equation (3) is corrected to determine the amount of compression damage sustained by wheat kernels after n consecutive compressions as follows:

(11) $$D_{n}^{*}={\left(\frac{\alpha -\alpha_{s}}{\alpha_{c}-\alpha_{s}}\right)}^{\gamma }$$

### 2.3. Continuous Damage Modeling of Wheat Kernels

Assuming that wheat kernels are approximately homogeneous ellipsoids exhibiting both elastic and elastic–plastic deformation behaviors under external loads, this study constructed a continuous extrusion damage model for wheat kernels. To elucidate the relationship between changes in normal load and compression deformations during the continuous extrusion process, this study referred to the ASAE S368.4 DEC2000 (R2017) standard and the elastic–plastic material model developed by Brizmer V et al. based on Hertz theory.

The equivalent radius *R_en_* of the wheat kernels at the nth compression is

(12) $$\frac{1}{R_{en}}=\frac{1}{R_{n}}+\frac{1}{R_{n}^{\prime }}$$

By substituting Equations (8) and (12) into the equation for determining the shape variable along the load direction in Hertz’s contact theory [26], the mechanical compression equation for wheat kernels during the elastic compression stage of the continuous compression process can be obtained as follows:

(13) $$F=\frac{\pi }{3}K^{-\frac{3}{2}}E_{n}^{*}R_{en}^{\frac{1}{2}}\alpha^{\frac{3}{2}}$$

By referring to the elastic–plastic material model developed by Brizmer V et al. [31], along with the formula for calculating the elastic modulus of wheat kernels during the continuous damage process (Equation (9)), and the formula for the mechanical compression equation of wheat kernels in the elastic compression stage (Equation (13)) established in this paper, the mathematical model relating the normal load *F* and the compression shape variable *α* for each compression during the continuous damage process can be determined as follows:

(14) $$F=\{\begin{array}{c} \frac{\pi }{3}K^{-\frac{3}{2}}E_{0}^{*}\overset{n}{\underset{i=1}{\prod }}\left(1-D_{i}^{*}\right)R_{en}^{\frac{1}{2}}\alpha^{\frac{3}{2}}\;\alpha^{\frac{3}{2}}⩽\alpha_{s}^{\frac{3}{2}},\;\;\mathrm{Elastic}\;\mathrm{stage}\\ F_{s}+E_{0}^{*}\overset{n}{\underset{i=1}{\prod }}\left(1-D_{i}^{*}\right)R_{en}^{\frac{1}{2}}\left(\alpha^{\frac{3}{2}}-\alpha_{s}^{\frac{3}{2}}\right)\;\alpha^{\frac{3}{2}}⩾\alpha_{s}^{\frac{3}{2}},\;\;\mathrm{Plastic}\;\mathrm{stage} \end{array}$$

## 3. Materials and Methods

To verify the accuracy of the previously constructed continuous damage model for wheat kernels, this study conducted continuous compression tests on both hard and soft wheat kernels. During these tests, the apparent damage at each stage—from the initial state to the point of crushing—was recorded. Experimental *F*–*α* curves were generated based on the data obtained, depicting the relationship between the normal load and the compression deformation for each compression stage. The characteristic parameters of the kernels after each compression-induced damage were calculated. Finally, the continuous damage process of the wheat kernels was analyzed, and the consistency between the actual damage evolution and the theoretical damage accumulation criterion was evaluated.

### 3.1. Test Materials and Instruments

**Figure 3:**
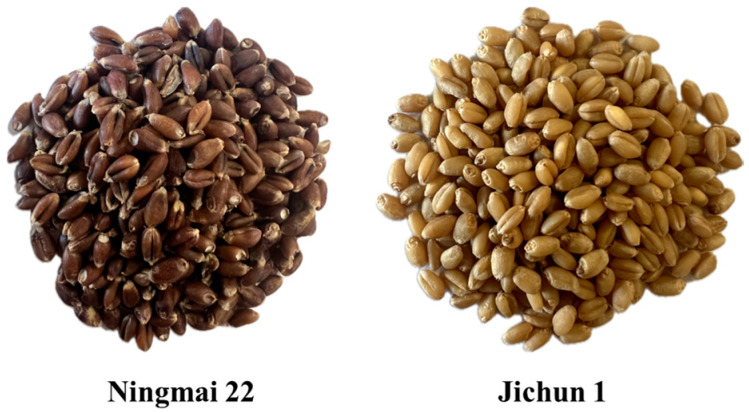
Testing materials.

**Figure 4:**
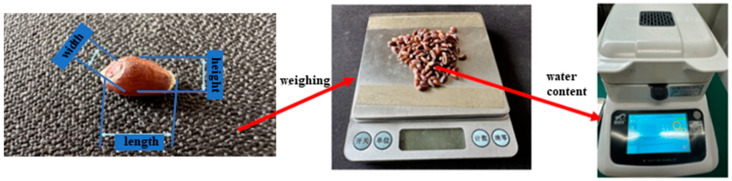
Test material measurement process.

As shown in Figure 3 and Figure 4, the test materials used in this study were Ningmai 22 (hard red wheat) from Xinyang, Henan Province, and Jichun 1 (soft white wheat) from Baoding, Hebei Province. The moisture content of the test samples was 12.5% and 12.8%, respectively, and the thousand-kernel weights were 70.20 g and 73.00 g, as per the national standard (GB/T 5497-1985) [32]. The experimental instruments included vernier calipers (precision: 0.01 mm); an electronic SQP balance (precision: 0.01 g); food-grade plastic measuring cups (300 mL); an XF-800MB moisture tester; a TA.XTC-18 mass spectrometer (detection error less than 0.015%); a Windows 10 system computer; a B011/Z008 Super Eyes electron microscope; and an R2890/LED ring light source.

### 3.2. Test Methods

#### 3.2.1. Sampling and Pre-Treatment

Kernels with a uniform and full appearance, free from obvious insect damage, deterioration, or injury, were randomly selected from each type of wheat as test samples. The triaxial dimensions of all kernels in the samples were measured, and the thickness was used to classify the kernels of each variety into three categories: small, medium, and large. The grain size (thickness) distributions for the wheat kernels were 2.58–3.08 mm for Ningmai 22 and 2.84–3.56 mm for Jichun 1. To reduce sampling error and exclude the largest and smallest grain sizes, which represented a very low percentage, the thicknesses of the small, medium, and large kernels were standardized as follows: Ningmai 22—2.67 ± 0.07 mm, 2.81 ± 0.07 mm, and 2.95 ± 0.07 mm; and Jichun 1—2.98 ± 0.11 mm, 3.20 ± 0.11 mm, and 3.42 ± 0.11 mm.

#### 3.2.2. Test Parameter Setting

Wheat kernels are approximately ellipsoidal in shape. According to the standard compression tool settings of ASAE S368.4 DEC2000 (R2008) [30], the probe model TA/5 was used with the following settings: pre-test speed of 60 mm/min, test loading speed of 1.2 mm/min, and post-test speed of 60 mm/min. The loading distance was set to 1 mm, with a trigger force of 0.1 N, and normal loading was applied along the direction of the kernel’s thickness. To establish a reasonable continuous compression loading distance, preliminary tests were conducted on different varieties and thicknesses of wheat kernels to determine the crushing deformation and normal load as indicators for a single loading test. The maximum compression deformation required to produce crushing was recorded. The results of the single loading tests showed that the average maximum deformation for small, medium, and large kernels of Ningmai 22 was less than 0.62 mm, 0.70 mm, and 0.77 mm, respectively. For Jichun 1, the average maximum deformation was less than 0.78 mm, 0.82 mm, and 0.85 mm, respectively. To improve testing efficiency, the continuous compression loading distances were set as follows: for Ningmai 22, it was 0.35 mm, 0.40 mm, 0.45 mm, 0.50 mm, 0.55 mm, 0.60 mm, 0.65 mm, 0.70 mm, 0.75 mm, and 0.80 mm; for Jichun 1, it was 0.45 mm, 0.50 mm, 0.55 mm, 0.60 mm, 0.65 mm, 0.70 mm, 0.75 mm, 0.80 mm, 0.85 mm, and 0.90 mm.

#### 3.2.3. Compression Testing, Image Acquisition, Post-Processing

**Figure 5:**
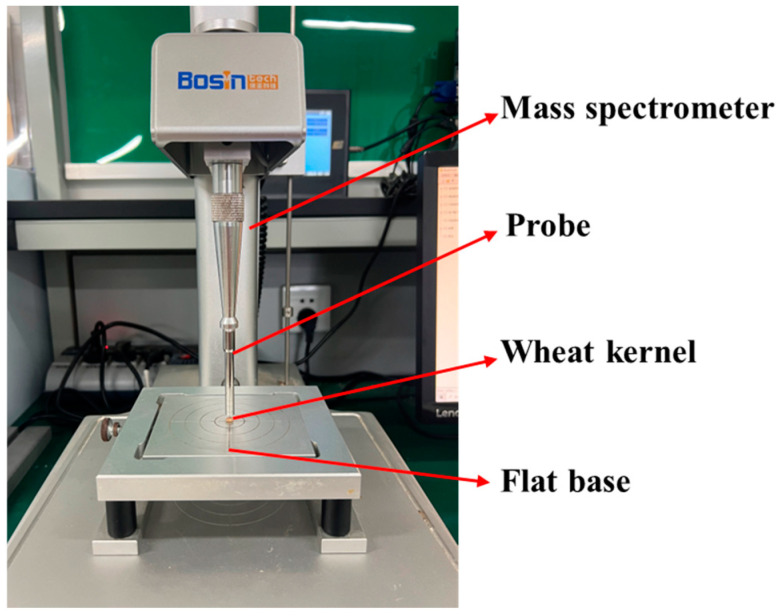
Continuous compression test setup.

**Figure 6:**
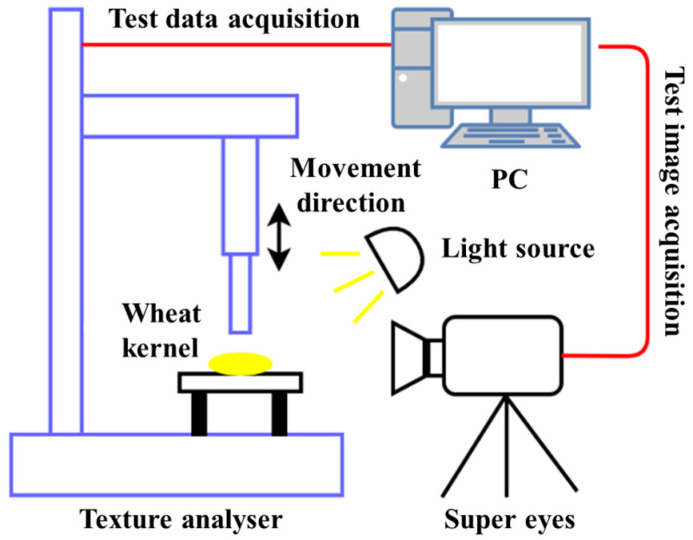
Test schematic diagram.

The entire testing process is illustrated in Figure 5 and Figure 6. For each loading distance, the amount of compression deformation, compression load, and number of compressions required to crush the kernels were used as indices. Multiple loading tests were conducted, with each test being repeated 20 times for each loading distance, wheat variety, and size to ensure accuracy. During testing, images of the visible changes in the wheat kernels, from successive damage to final breakage, were recorded using a Super Eyes electron microscope. At the end of each test, the number of compressions needed to crush the kernels was recorded for each loading distance, variety, and size. If the continuous loading was performed more than 30 times without causing kernel breakage, it was assumed that further damage would not occur. The average results from the 20 tests were used to determine the continuous compression and crushing outcomes. The experimental data were then exported and plotted to show the relationship between normal load and compression deformation throughout the continuous damage process. Additionally, the elastic modulus values after the initial compression and subsequent compressions were calculated (*E*_0_*, *E*_1_*, *E*_2_*, *E*_3_*, ……, E_n_*).

#### 3.2.4. Calculation of the Modulus of Elasticity after Continuous Compression of Test Specimens

The average values of the equivalent radius (*R_en_*) and the coefficient (*K_n_*) for each test sample after each compression were calculated using Equations (4)–(6) and (13). According to Equation (14), during the elastic compression stage, the relationship between the normal load (*F*) and the compression deformation (*α*^3/2^) is linear. Based on Hertz contact theory, the linear part of the compression change curve was used to approximate the values of *F* and *α*. These values, along with the previously calculated *R_en_* and *K_n_* values, were substituted into Equations (7) and (8) for further calculations. The average values obtained from 20 repeated tests were then used to determine the elastic modulus of the wheat kernels (E_n_*) after the nth compression.

## 4. Results and Discussion

### 4.1. Analysis of Continuous Compression Test Results

**Figure 7:**
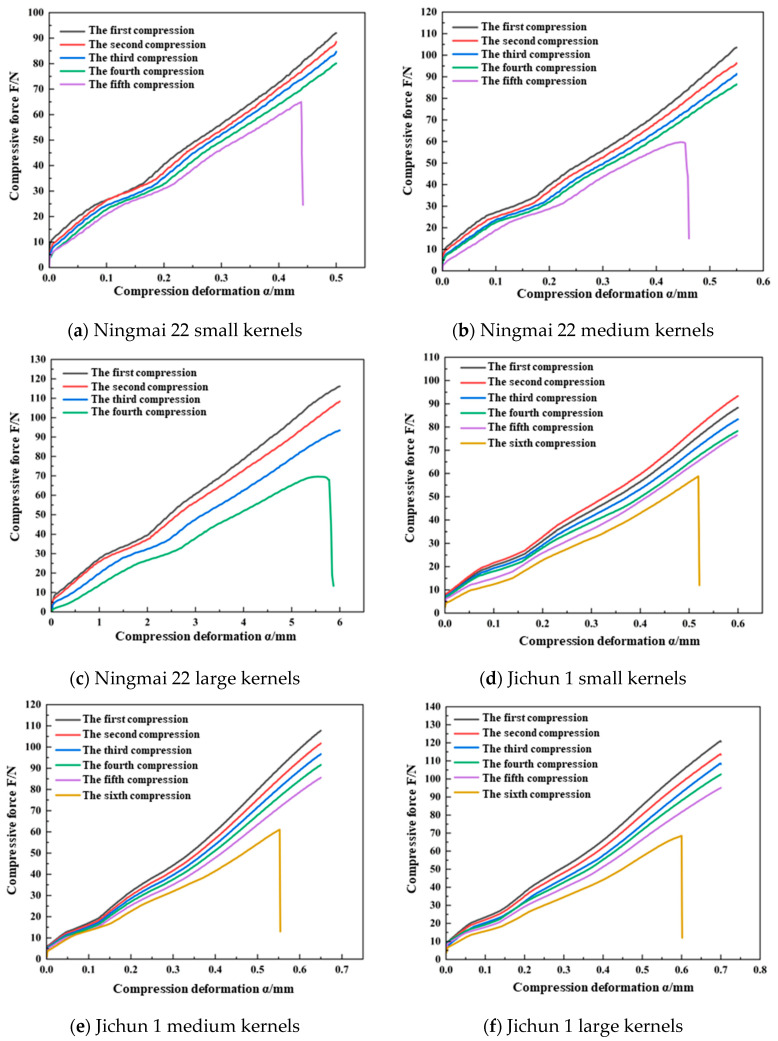
Continuous damage *F*–*α* curves of wheat kernels.

The results of the continuous compression test for the Ningmai 22 kernels are summarized in Table 1. The initial deformation intervals for small, medium, and large kernels were 0 mm to 0.35 mm. Within this range, applying 30 consecutive mechanical compressions did not lead to kernel fragmentation. However, when the deformation intervals were increased to 0.35 mm to 0.60 mm, 0.35 mm to 0.65 mm, and 0.35 mm to 0.75 mm, continuous application of the compression load resulted in kernel fragmentation. Notably, the number of consecutive compressions required to cause fragmentation decreased with an increasing deformation interval. For deformation amounts exceeding 0.60 mm, 0.65 mm, and 0.75 mm, a single compression load was sufficient to directly cause fragmentation. As examples, the variation curves of the normal load with the compression set for small kernels (0.50 mm), medium kernels (0.55 mm), and large kernels (0.60 mm) during the entire process of successive damage are illustrated in Figure 7a, Figure 7b, and Figure 7c, respectively.

The results of the continuous compression test for the Jichun 1 kernels are summarized in Table 1. The initial deformation intervals for small, medium, and large kernels were 0 mm to 0.45 mm, 0 mm to 0.45 mm, and 0 mm to 0.50 mm, respectively. Within these ranges, applying 30 consecutive mechanical compressions did not result in kernel fragmentation. However, when the deformation intervals were increased to 0.45 mm to 0.70 mm, 0.45 mm to 0.80 mm, and 0.50 mm to 0.85 mm, the continuous application of compression loads led to kernel fragmentation. The number of consecutive compressions required to cause fragmentation decreased as the deformation interval increased. For deformation amounts exceeding 0.70 mm, 0.80 mm, and 0.85 mm, a single compression load was sufficient to directly cause particle crushing. For example, the curves showing the variation in the normal load with the compression deformation variable for small kernels with deformation variables of 0.60 mm and 0.75 mm, medium kernels with deformation variables of 0.65 mm and 0.85 mm, and large kernels with deformation variables of 0.70 mm and 0.90 mm are illustrated in Figure 7d, Figure 7e, and Figure 7f, respectively.

The reasons for the above results are as follows: the compression and deformation process of wheat kernels generally occurs in three stages: the elastic compression stage, the plastic compression stage, and the intensive destruction stage. In the initial stage of deformation, when the compression remains within the elastic–plastic threshold, the wheat kernels undergo elastic compression. At this stage, the internal structure of the kernels remains unchanged under mechanical compression, and they retain their elasticity. Upon removal of the external load, the wheat kernels naturally return to their original shape without sustaining damage.

When the deformation caused by compression exceeds the elastic–plastic threshold, the kernels enter the plastic compression stage. In this stage, continued mechanical compression induces microscopic defects within the kernels. As the number of compression cycles increases, these defects expand and merge, ultimately leading to kernel fracture. The greater the compression deformation at this stage, the faster the defects propagate, resulting in fewer compression cycles before the kernels break.

Finally, when the deformation surpasses the maximum crushing limit, the wheat kernels reach the stage of intensified destruction. At this point, a single compression is sufficient to cause rapid initiation, expansion, and fusion of internal damage, which becomes visible on the surface as the kernels are crushed.

Combining the above analysis with the results of the study by Yuan Jiacheng et al. [23] on the continuous damage crushing process of rapeseed kernels, which share similar elastic–plastic properties with wheat kernels, it is evident that the critical value of elastic–plastic deformation and the maximum crushing deformation are key parameters for analyzing the behavior of kernels during each stage of the continuous damage process. The performance of elastic–plastic kernels across the elastic compression, plastic compression, and intensive destruction stages remains consistent. However, due to variations in kernel type, size, moisture content, and other intrinsic attributes, the critical value of elastic–plastic deformation and the maximum crushing deformation differ significantly. Consequently, the size of the deformation zones in each of these three stages also varies.

### 4.2. Analysis of Continuous Compression Crushing Process of Wheat Kernels

The parameters of the compression crushing results for the wheat kernels are detailed in Table 2 and Table 3. The successive damage *F*–*α* curves for each wheat variety and size revealed that the overall relationship between the normal load and compression deformation was consistent across the different samples. Specifically, the curves exhibited a similar trend, with the slope changing once the compression deformation reaches the elastic–plastic deformation threshold. Additionally, as the number of consecutive compressions increased, the maximum normal load observed during kernel unloading decreased continuously.

When Ningmai 22 and Jichun 1 were subjected to continuous compression, the values of the equivalent radius (*R_en_*) and contact stiffness (*K_n_*) increased with the number of compressions. This phenomenon occurs because continuous axial mechanical compression causes the deformation of the wheat kernels to exceed the critical elastic–plastic threshold. As a result, internal damage accumulates within the kernels, which is reflected in a numerical decrease in the elastic modulus (E_n_*). As the axial thickness of the kernels gradually decreases, their ability to recover from deformation diminishes. Simultaneously, the contact area during compression increases, leading to a corresponding rise in the equivalent radius (*R_en_*) and contact stiffness (*K_n_*).

**Figure 8:**
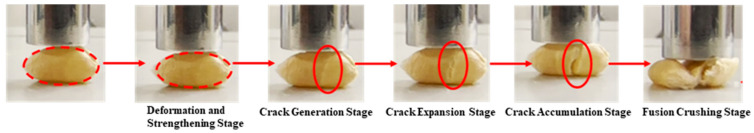
Images of the whole process of continuous damage to wheat kernels.

Figure 8 illustrates the entire process of continuous damage and crushing of wheat kernels. Throughout this process, the wheat kernels undergo repeated compression, leading to various stages of damage [24,33]. The damage process can be categorized into the following stages:

(1) Small Deformation Stage: The initial compression causes minimal deformation with no significant damage.
(2) Deformation and Strengthening Stage: As the compression continues, the kernels deform and undergo strengthening due to the applied pressure.
(3) Crack Generation Stage: Micro-cracks start to form within the kernels as the deformation exceeds the elastic limit.
(4) Crack Expansion Stage: The existing cracks begin to expand under the continued pressure.
(5) Crack Accumulation Stage: Cracks accumulate and grow, leading to significant structural damage.
(6) Fusion Crushing Stage: The accumulation of cracks and continued pressure result in the complete crushing and fragmentation of the kernels.

To determine the critical value of elastic–plastic deformation (*α_s_*) for wheat kernels of various varieties and sizes, the steps were as follows:

(1) Calculate the Elastic Modulus: Use the previously described method to compute the modulus of elasticity (E_n_*) of the wheat kernels for each compression. This involves measuring the modulus of elasticity after each compression event during the continuous compression process.
(2) Obtain the Mechanical Compression Function Curve: With the calculated elastic modulus values, substitute these into Equation (14) to generate the mechanical compression function curve. This curve represents the relationship between the normal load and the compression deformation for each compression stage.
(3) Determine the Critical Elastic–Plastic Deformation (*α_s_*): Compare the mechanical compression function curve with the continuous damage F–α curve obtained from experimental tests. The intersection point of these two curves represents the critical value of elastic–plastic deformation (*α_s_*) for the wheat kernels.
(4) Compile Material Property Parameters: Gather and record the initial elastic modulus and other relevant material property parameters for the wheat kernels under continuous compression, as shown in Table 3.

From Equation (13), the relationship between *F* and *α*^3/2^ is linear. To simplify the compression characteristic parameters of the continuous damage process of wheat kernels, the coefficient *M* is used for their definition:

(15) $$M=\frac{\pi }{3}K^{-\frac{3}{2}}E_{n}^{*}R_{en}^{\frac{1}{2}}$$

By substituting the compression characteristic parameters from the continuous compression process of wheat kernels into Equation (15), the average mechanical compression function equation coefficients *M* for Ningmai 22 small, medium, and large kernels were 263.75, 289.71, and 311.17, respectively, while for Jichun 1 small, medium, and large kernels, the coefficients were 213.05, 226.93, and 263.75, respectively. Consequently, the mechanical compression equations for the wheat kernels were as follows:

(16) $$\{\begin{array}{c} F_{Ns}=263.75\alpha^{\frac{3}{2}}\\ F_{Nm}=289.71\alpha^{\frac{3}{2}}\\ F_{Nl}=311.17\alpha^{\frac{3}{2}} \end{array}$$

(17) $$\{\begin{array}{c} F_{Js}=213.05\alpha^{\frac{3}{2}}\\ F_{Jm}=226.93\alpha^{\frac{3}{2}}\\ F_{Jl}=263.75\alpha^{\frac{3}{2}} \end{array}$$

Based on the above mechanical compression function curves of wheat kernels and the continuous damage *F*–*α* curves obtained from the tests, the compression and crushing data for each variety and size of wheat kernels were summarized, as shown in Table 4.

### 4.3. Continuous Compression Characterization

Based on the results of the continuous compression test on the wheat kernels, it was observed that if the deformation caused by the compression load is below the critical threshold for elastic–plastic deformation, continued loading does not result in kernel crushing. However, if the deformation exceeds this critical threshold but remains below the maximum crushing deformation, the continuous load will lead to kernel crushing. In this scenario, the number of compression cycles and the degree of kernel deformation are inversely related. Conversely, if the deformation surpasses the maximum crushing deformation, a single load will cause immediate kernel crushing. Additionally, if the number of consecutive loads exceeds the maximum crushing deformation, a single load will directly lead to kernel crushing.

Taking the small kernels of Ningmai 22 and Jichun 1 as examples, tests were conducted with compression deformation variables of 0.50 mm and 0.60 mm and compression cycles of 5 and 6, respectively (Figure 3a,d). The parameters obtained from the continuous compression tests are summarized in Table 5 and Table 6.

As shown in Table 5 and Table 6, the mean values of the elastic–plastic critical deformation for the Ningmai 22 and Jichun 1 kernels in each successive compression were 0.37 mm and 0.45 mm, respectively. These values align with the damage limit ranges presented in Table 1, specifically 0.35 mm to 0.40 mm and 0.45 mm to 0.50 mm. The elastic–plastic critical force in each compression cycle exhibited an increasing trend as the number of compressions increased, while the critical deformation remained relatively stable. Figure 7a,d and Table 5 and Table 6 demonstrate that the *F_s_*–*α_s_*^3/2^ ratio and the equivalent radius (*R_en_*) of the wheat kernels tended to increase with successive compressions, whereas the integrated modulus of elasticity (E_n_*) showed a declining trend. According to the continuous damage theory and the wheat kernel model described in the previous section, when the compression deformation surpasses the critical value of elastic–plastic deformation, irreversible damage occurs in the wheat kernels. With continued compression, this damage accumulates, leading to an increase in kernel deformation and a corresponding deterioration in mechanical properties. This degradation is reflected numerically by a significant increase in the equivalent radius (*R_en_*) and a decrease in the integrated elastic modulus (E_n_*), with the rate of increase in Ren notably outpacing the rate of decrease in E_n_* [23,24,25].

Analyzing the above results of the internal microscopic changes in the kernels, it is clear that wheat kernels are not uniform isotropic bodies; they contain micro-defects, such as pore spaces. As the external load and compression increase, these micro-defects expand and merge, causing damage to the original pore structure. The pores shrink and become denser, resulting in a gradual increase in the elastic–plastic critical force of the kernels as the number of compressions rises. Since the elastic–plastic critical deformation is an inherent property of the kernels—dependent only on factors like variety, size, and moisture content, and unaffected by external conditions—it did not show significant changes in the test results.

Referring to Yuan Jiacheng et al.’s study on the continuous damage characteristics of rapeseed kernels, it was observed that during the nine consecutive compression cycles of Huayouza 62 rapeseed kernels, the elastic–plastic critical force gradually increased from the initial 7.94 N to the final crushing force of 8.76 N. Throughout these compression cycles, the elastic–plastic critical deformation remained stable at 0.33 mm, without any significant variation. While wheat kernels and rapeseed kernels differ in shape, size, and variety, both can be regarded as non-homogeneous elastic–plastic bodies. Thus, the comprehensive elastic modulus and equivalent radius of wheat kernels obtained in this experiment align closely with the continuous damage progression observed in rapeseed kernels, despite noticeable differences in the parameters measured in the experiments of Yuan Jiacheng and others [23]. In addition, Man Xiaolan et al. also used force–deformation curve analysis to describe the changes in equivalent radius and integrated elastic modulus in walnuts subjected to a continuous damage process, and obtained the size of the critical value parameter that determines the fracture mechanism of walnuts. The change rule of the above experimental parameters is basically consistent with the previous analysis [25].

Considering that smaller wheat kernels are more susceptible to breakage during processing, and based on the results of the continuous compression tests presented in Table 5 and Table 6, the following parameters are recommended for wheat peeling machines.

For processing Ningmai 22 kernels, the peeling pressure should be set to less than 62.48 N, which is the average elastic–plastic critical pressure of the kernels. Additionally, the deformation amount should be kept below 0.37 mm, the average elastic–plastic critical deformation for these kernels.

For processing Jichun 1 kernels, the peeling pressure should be maintained at less than 58.76 N, the average elastic–plastic critical pressure of these kernels. Similarly, the deformation amount should be restricted to below 0.45 mm, the average elastic–plastic critical deformation for Jichun 1 kernels.

**Figure 9:**
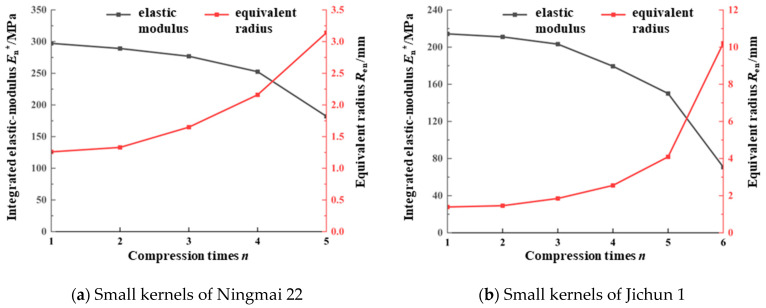
Variation curves of integrated modulus of elasticity and equivalent radius.

The variation curves of the integrated modulus of elasticity (E_n_*) and the equivalent radius (*R_en_*) for Ningmai 22 and Jichun 1 under continuous compression are illustrated in Figure 9.

Figure 9 illustrates that the integrated modulus of elasticity (E_n_*) of the wheat kernels of all varieties decreased progressively with an increasing number of compressions (*n*). Specifically, Ningmai 22 and Jichun 1, which underwent 5 and 6 compressions, respectively, experienced crushing. At the final compression, the integrated modulus of elasticity (E_n_*) of Ningmai 22 and Jichun 1 was reduced to 3/5 and 1/3 of the initial value, respectively. Conversely, the equivalent radius (*R_en_*) of the wheat kernels of all varieties increased with the number of compressions, following a nearly quadratic growth curve. At the final compression, the equivalent radius (*R_en_*) of Ningmai 22 and Jichun 1 increased to 5/2 and 15/2 times their initial values, respectively. These results are consistent with the previous theoretical analysis and support the notion that the continuous compression damage evolution of wheat grains conforms to the linear cumulative damage criterion proposed by continuous damage theory.

### 4.4. Continuous Damage Model Validation

To further validate the accuracy of the linear cumulative damage theory for wheat kernels proposed in this study, the developed continuous damage model was subjected to validation.

**Figure 10:**
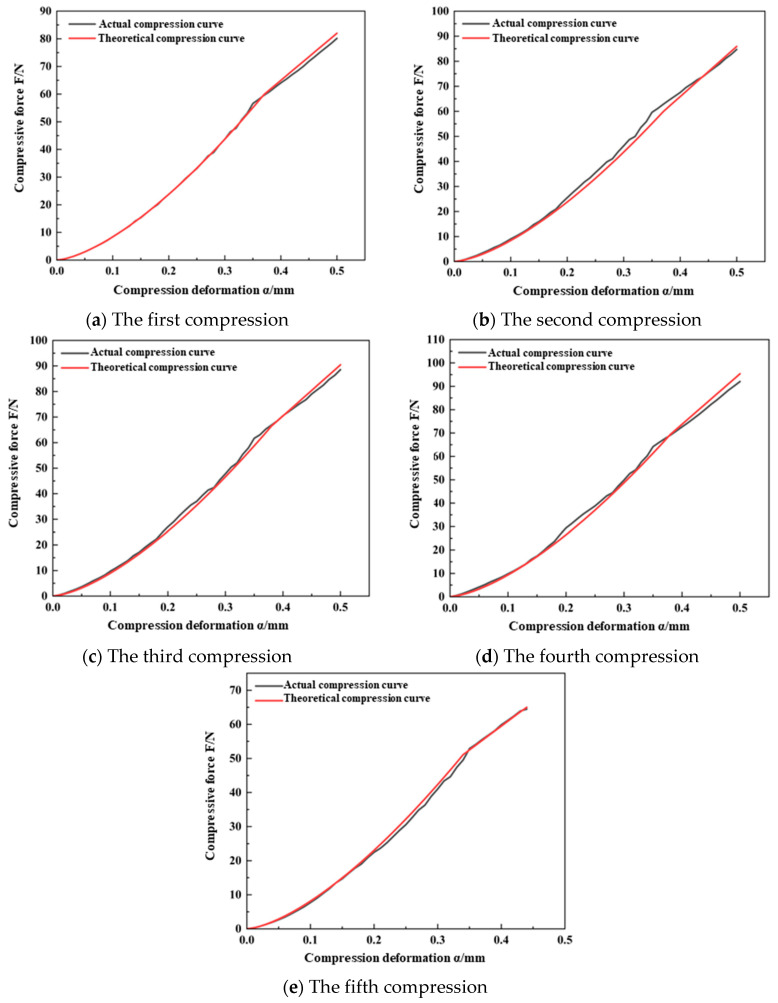
The actual and theoretical curves of continuous compression of Ningmai 22.

**Figure 11:**
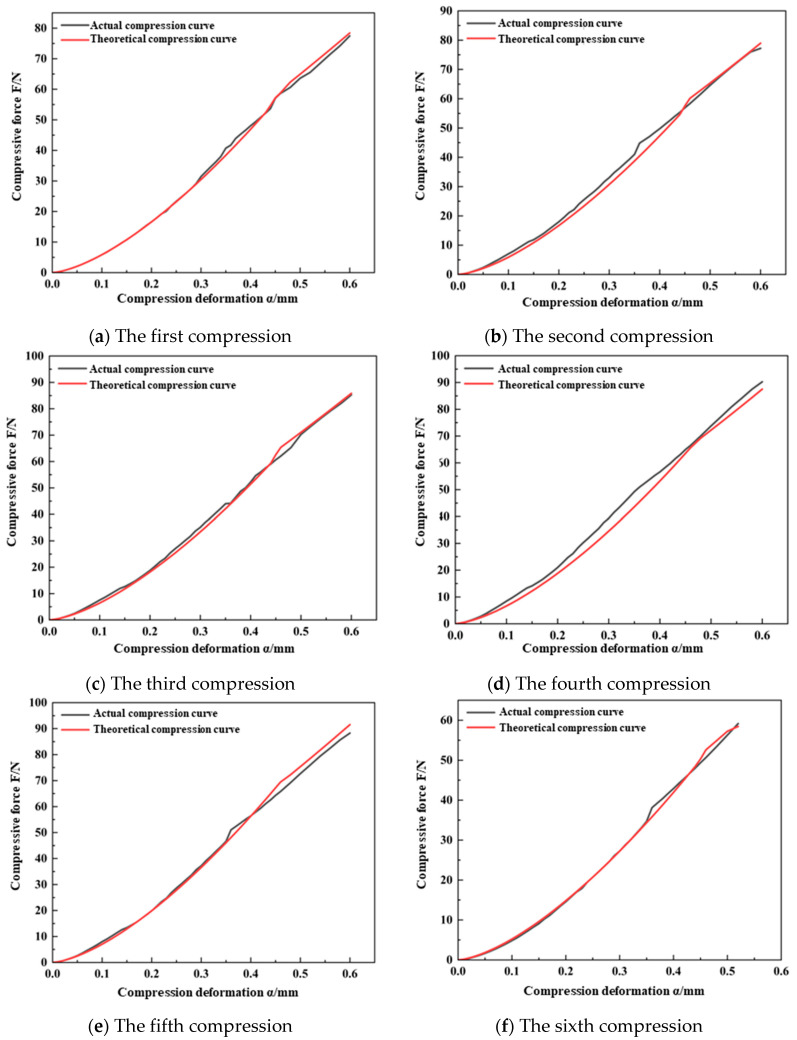
The actual and theoretical curves of continuous compression of Jichun 1.

Substituting the results from the continuous compression crushing tests of the wheat kernels, as detailed in Table 5 and Table 6, into Equation (9), the damage variables (*D*) were calculated as follows: for Ningmai 22 kernels, the values were 0.027, 0.042, 0.088, and 0.279; for Jichun 1 kernels, the values were 0.015, 0.037, 0.117, 0.164, and 0.529. By incorporating these results into the continuous damage model for wheat kernels, as described by Equation (15), the *F*–*α* theoretical curves for Ningmai 22 and Jichun 1 at each compression stage in the damage process were obtained. These theoretical compression curves were then compared and fitted against the actual compression curves to assess the accuracy of the continuous damage model developed in this study, as illustrated in Figure 10 and Figure 11.

The results shown in Figure 10 and Figure 11 indicate that the elastic–plastic critical deformation variable (α*_s_*) of Ningmai 22 and Jichun 1 stabilized at around 0.35 mm and 0.45 mm, respectively, during each compression cycle. This behavior aligns with the trend observed in the previous analysis of the continuous damage of wheat kernels. Additionally, the overall slope of the actual compression curves exhibited a noticeable decreasing trend, which corresponds well with the theoretical analysis of the comprehensive elastic modulus (E_n_*) and equivalent radius (*R_en_*).

To further compare the actual compression curves of Ningmai 22 and Jichun 1 with their theoretical counterparts, the maximum deviations between the actual and theoretical curves were calculated. The ratio of these deviations to the maximum normal load during crushing was determined, and the goodness-of-fit (*R*²) of the compression curves was also evaluated. For Ningmai 22, the maximum deviations of the actual normal load from the theoretical load during each compression were 2.14 N, 4.52 N, 2.93 N, 3.36 N, and 2.17 N, respectively. For Jichun 1, the maximum deviations were 2.31 N, 4.43 N, 3.31 N, 4.98 N, 3.46 N, and 2.98 N, respectively. In all cases, the deviations accounted for less than 5% of the crushing normal load, and the *R*² values for the curve fitting exceeded 0.9.

In conclusion, the continuous damage model for wheat kernels developed in this study demonstrated a certain degree of accuracy.

## 5. Conclusions and Outlook

The following conclusions can be drawn:

1. In this study, a continuous damage model for wheat kernels was developed, and equations were derived to describe the relationship between the normal load and compression deformation variables under continuous damage conditions.
2. The results of the continuous compression crushing of wheat kernels show that the average maximum crushing force *F*_c_ of small-, medium-, and large-sized kernels of Ningmai 22 was 96.71 ± 2.27 N, 110.17 ± 2.68 N, and 128.41 ± 2.85 N, respectively; the average maximum crushing deformation *α_c_* was 0.65 ± 0.08 mm, 0.68 ± 0.13 mm, and 0.77 ± 0.17 mm, respectively; and the average critical value of elastic–plastic deformation *α_s_* was 0.37 mm, 0.38 mm, and 0.39 mm, respectively. The average maximum crushing force *F*_c_ of small-, medium-, and large-sized kernels of Jichun 1 was 113.37 mm, 0.38 mm, and 0.39 mm, respectively. The average maximum crushing force *F_c_* of small-, medium-, and large-sized Jichun 1 kernels was 113.34 ± 3.15 N, 125.28 ± 3.64 N, and 136.15 ± 3.29 N, respectively; the average maximum crushing deformation *α_c_* was 0.75 ± 0.11 mm, 0.83 ± 0.15 mm, 0.88 ± 0.15 mm, 0.88 ± 0.17 mm, and 0.85 ± 0.85 N, respectively; the average maximum crushing deformation *α_c_* was 0.65 ± 0.08 mm, 0.68 ± 0.13 mm, 0.77 ± 0.17 mm, 0.15 mm, and 0.88 ± 0.18 mm, respectively; and the average critical values of elastic–plastic deformation *α_s_* were 0.45 mm, 0.47 mm, and 0.52 mm, respectively.
3. When the compression deformation of the wheat kernels was below the critical damage threshold, the continuous application of a compression load did not result in breakage. However, when the compression deformation exceeded this critical threshold, continued compression led to kernel breakage. Since smaller-sized wheat kernels are more prone to breakage during actual processing, it is essential to control the peeling pressure (*F*) and the deformation (*α*) generated by the compression in the wheat peeling machine to be lower than the average maximum crushing force (*F_c_*) and the average elastic–plastic critical deformation (*α_s_*) of the smaller-sized wheat kernels.

In the actual peeling process, the compression contact orientation of wheat kernels exhibits dynamic changes due to interactions with the rolling peeling plate and between kernels. Additionally, there is currently no standardized reference for the pre-peeling wetting treatment of different wheat varieties. Considering that wheat kernels with varying moisture contents may exhibit different compression characteristics under continuous damage, it is important to conduct continuous damage tests based on the model developed in this study. Such tests should analyze the effects of compression orientation and moisture content on the parameters of continuous damage. This will help refine the model and provide theoretical insights into the mechanisms of damage and fragmentation of wheat kernels under continuous mechanical action.

## Figures and Tables

**Table 1:** Multiple compression crushing results for wheat kernels.

Variety	Size	Compression Deformation *α*/mm											
		0.35	0.40	0.45	0.50	0.55	0.60	0.65	0.70	0.75	0.80	0.85	0.90
Ningmai 22	Small	>30	12.6	7.3	5.4	3.1	1.7	1.0	1.0	1.0	1.0	1.0	1.0
	Medium	>30	17.3	12.2	7.8	5.3	2.7	1.5	1.0	1.0	1.0	1.0	1.0
	Large	>30	26.8	20.4	14.5	8.6	4.9	3.2	2.1	1.3	1.0	1.0	1.0
Jichun 1	Small	>30	>30	>30	16.7	12.4	6.3	2.9	1.3	1.0	1.0	1.0	1.0
	Medium	>30	>30	>30	22	14.5	11.5	5.7	1.6	1.5	1.3	1.0	1.0
	Large	>30	>30	>30	>30	21.6	14.7	9.2	6.1	4.5	2.8	1.4	1.0

**Table 2:** Continuous compression crushing parameters for Ningmai 22.

Compression Cycles *n*	Small Kernels			Medium Kernels			Large Kernels		
	Equivalent Radius *R_en_*/mm	*K_n_*	Elastic Modulus E_n_*/MPa	Equivalent Radius *R_en_*/mm	*K_n_*	Elastic Modulus E_n_*/MPa	Equivalent Radius *R_en_*/mm	*K_n_*	Elastic Modulus E_n_*/kPa
1	1.257	1.198	297.240	1.422	1.235	318.472	1.576	1.267	337.629
2	1.328	1.267	289.10	1.502	1.293	308.918	1.664	1.314	327.513
3	1.651	1.314	276.88	1.867	1.331	293.472	2.069	1.342	311.137
4	2.163	1.342	252.52	2.445	1.349	267.059	2.710	1.349	283.135
5	3.139	1.351	182.05	3.548	1.351	192.28	3.932	1.351	203.857

**Table 3:** Continuous compression crushing parameters for Jichun 22.

Compression Cycles *n*	Small Kernels			Medium Kernels			Large Kernels		
	Equivalent Radius *R_en_*/mm	*K_n_*	Elastic Modulus E_n_*/MPa	Equivalent Radius *R_en_*/mm	*K_n_*	Elastic Modulus E_n_*/MPa	Equivalent Radius *R_en_*/mm	*K_n_*	Elastic Modulus E_n_*/kPa
1	1.390	1.267	214.31	1.543	1.293	256.545	1.691	1.235	265.872
2	1.463	1.293	211.06	1.625	1.314	252.697	1.781	1.293	261.884
3	1.848	1.314	203.21	2.052	1.331	243.347	2.249	1.331	252.194
4	2.553	1.331	179.44	2.834	1.342	214.875	3.106	1.342	222.687
5	4.092	1.342	150.04	4.543	1.349	179.636	4.979	1.349	186.166
6	10.214	1.351	70.68	11.339	1.351	84.428	12.427	1.351	87.498

**Table 4:** Test data for wheat kernel compression crushing.

Variety	Size	Average Maximum Crushing Force *F_c_*/N	Average Maximum Crushing Deformation *α_c_*/mm	Elastic–Plastic Critical Average Pressure *F_s_*/N	Average Critical Value of Elastic–Plastic Deformation *α_s_*/mm
Ningmai 22	Small	96.71 ± 2.27	0.65 ± 0.08	50.21	0.37
	Medium	110.17 ± 2.68	0.68 ± 0.13	60.13	0.38
	Large	128.41 ± 2.85	0.77 ± 0.17	59.08	0.39
Jichun 1	Small	113.34 ± 3.15	0.75 ± 0.11	58.11	0.45
	Medium	125.28 ± 3.64	0.83 ± 0.15	64.17	0.47
	Large	136.15 ± 3.29	0.88 ± 0.18	85.05	0.52

**Table 5:** The results of continuous compression test of Ningmai 22 (small kernels).

Compression Cycles *n*	1	2	3	4	5
Elastic–plastic critical pressure *F_s_*/N	60.63	61.87	66.72	70.26	52.91
Elastic–plastic critical deformation *α_s_*/mm	0.373	0.378	0.381	0.383	0.348
*F_s_/α_s_* ^3/2^	266.08	266.17	283.65	296.37	257.68
Equivalent radius *R_en_*/mm	1.26	1.33	1.65	2.16	3.14
Integrated elastic modulus E_n_*/MPa	297.24	289.10	276.88	252.52	182.05

**Table 6:** The results of continuous compression test of Jichun 1 (small kernels).

Compression Cycles *n*	1	2	3	4	5	6
Elastic–plastic critical pressure *F_s_*/N	52.67	55.01	58.74	66.33	70.38	49.40
Elastic–plastic critical deformation *α_s_*/mm	0.432	0.442	0.437	0.463	0.464	0.446
*F_s_/α_s_* ^3/2^	185.46	187.17	203.30	210.50	222.63	165.82
Equivalent radius *R_en_*/mm	1.39	1.46	1.85	2.55	4.09	10.21
Integrated elastic modulus E_n_*/MPa	214.31	211.06	203.21	179.44	150.04	70.68

## Data Availability

The original contributions presented in the study are included in the article, further inquiries can be directed to the corresponding author.
